# Native Hawaiian/Pacific Islander alcohol, tobacco and other drug use, mental health and treatment need in the United States during COVID‐19

**DOI:** 10.1111/dar.13522

**Published:** 2022-08-11

**Authors:** Andrew M. Subica, Erick G. Guerrero, Tammy K. K. Martin, Scott K. Okamoto, Nia Aitaoto, Howard B. Moss, Brittany N. Morey, Li‐Tzy Wu

**Affiliations:** ^1^ School of Medicine University of California Riverside USA; ^2^ Research to End Healthcare Disparities Corp I‐Lead Institute Los Angeles USA; ^3^ School of Social Work Hawai‘i Pacific University Honolulu USA; ^4^ Pacific Islander Center of Primary Care Excellence San Leandro USA; ^5^ Department of Health, Society, and Behavior University of California Irvine USA; ^6^ School of Medicine Duke University Durham USA

**Keywords:** alcohol use disorder, alcohol, tobacco, drug use, COVID‐19, mental health, Native Hawaiian/Pacific Islanders

## Abstract

**Introduction:**

Before COVID‐19, Native Hawaiians/Pacific Islanders (NH/PI) endured a heavy burden of alcohol, tobacco and other drug (ATOD) use in prior US data. Responding to reports that many NH/PI communities experienced severe COVID‐19 disparities that could exacerbate their ATOD burden, we partnered with NH/PI communities to assess the substance use patterns and treatment needs of diverse NH/PIs during COVID‐19.

**Methods:**

Collaborating with NH/PI community organisations across five states with large NH/PI populations, we conducted a large‐scale investigation of NH/PI ATOD use, mental health and treatment need during COVID‐19. Between April and November 2021, NH/PI‐heritage research staff from our community partners collected data involving 306 NH/PI adults using several community‐based recruitment methods (e‐mail, telephone, in‐person) and two survey approaches: online and paper‐and‐pencil. Multivariate regressions were conducted to examine potential predictors of NH/PI alcohol use disorder and need for behavioural health treatment.

**Results:**

During COVID‐19, 47% and 22% of NH/PI adults reported current alcohol and cigarette use, while 35% reported lifetime illicit substance use (e.g., cannabis, opioid). Depression and anxiety were high, and alcohol use disorder, major depression and generalised anxiety disorder prevalence were 27%, 27% and 19%, respectively. One‐third of participants reported past‐year treatment need with lifetime illicit substance use, COVID‐19 distress and major depression respectively associating with 3.0, 1.2, and 5.3 times greater adjusted odds for needing treatment.

**Conclusions:**

NH/PI adults reported heavy ATOD use, depression, anxiety and treatment need during COVID‐19. Targeted research and treatment services may be warranted to mitigate COVID‐19's negative behavioural health impact on NH/PI communities.

## 1 INTRODUCTION

In the United States, Native Hawaiians/Pacific Islanders (NH/PI) are an understudied racial/ethnic group that has been deeply affected by COVID‐19. According to limited county and state data, NH/PI adults possess among the nation's highest rates of COVID‐19 infection, hospitalisation and mortality [1, 2]. This is in part due to several factors that increase NH/PIs' risk for SARS‐CoV‐2 exposure including: (i) employment in essential frontline positions (e.g., service industry, meatpacking factories); (ii) dwelling in dense households/neighbourhoods; and (iii) traditional sociocultural practices/obligations that necessitate large in‐person group contact (e.g., weddings, funerals) [3, 4]. Collectively, these factors limit COVID‐19 protective behaviours—including many recommended by the US government such as social distancing, restrictions on large group gatherings and indoor mask wearing—while enhancing the likelihood of ‘superspreader’ events in NH/PI communities [5, 6].

These risk factors combined with NH/PIs' pre‐existing disparities in obesity, diabetes, smoking, cardiovascular disease and cancer [7, 8, 9] have led to severe COVID‐19 outcomes (e.g., hospitalisations, deaths) that have ripped through many NH/PI communities [10]. In 2021, Penaia et al. [11] reported that NH/PIs possessed the highest per capita death rate in 90% of states reporting NH/PI COVID‐19 deaths. Yet, despite these extreme levels of COVID‐19 illness and death—and resulting trauma, loss and grief [12, 13]—no known studies have partnered with NH/PI communities to identify their behavioural health needs during COVID‐19.

This lack of targeted research closely parallels the glaring gaps in NH/PI research that have impacted NH/PIs for decades [14]. This is particularly evident in how little is known about NH/PI substance use and mental health, with the sparse existing literature suggesting that NH/PIs were affected by significant disparities in alcohol, tobacco and other drug (ATOD) use and mental disorders before COVID‐19. Accordingly, we postulated that during COVID‐19, NH/PI adults would demonstrate elevated ATOD use, mental health issues and treatment need [15].

### 1.1 NH/PI ATOD use

In prior data, Native Hawaiian youth displayed the highest rates of alcohol use, chronic drinking and binge drinking of all Hawai‘i youth [16, 17] while nationally, NH/PI youth reported among the highest prevalence of heavy episodic drinking and early alcohol use of any US racial/ethnic group between 1991 and 2015 [18]. Among adults, Hawai‘i data indicated that NH/PIs reported higher age‐adjusted rates of heavy episodic drinking versus Whites while recent community‐partnered studies [19] revealed NH/PI adults experienced alcohol use disorders (AUD) at rates four times greater than the 5% US population rate in 2019 [20]. Similarly, combustible cigarette smoking appears elevated among NH/PIs with up to 31% of NH/PI adults and 52% of young adults (18–30 years) smoking cigarettes [21, 22, 23] versus 14% of US adults in 2019 [24].

Concurrently, illicit cannabis, opioid and methamphetamine use remains underexamined in NH/PI communities despite opioids and methamphetamines representing: (i) the leading causes of US drug overdose deaths [25, 26]; and (ii) the subject of growing NH/PI community concerns. In 2021, public reports revealed grave community concern regarding Hawai‘i's rising opioid overdose epidemic [27, 28, 29, 30] while methamphetamine use has long posed a major concern for NH/PI communities and a leading cause of drug overdose deaths in states with significant NH/PI populations [26]. This includes Hawai‘i, where the rate of annual methamphetamine overdose deaths increased fourfold from 2009 to 2018 [31]. Yet, despite these community‐expressed concerns, US NH/PI substance use data remains largely unavailable.

### 1.2 Mental health in NH/PIs

Scant research also exists regarding NH/PI mental health with the few published studies suggesting high prevalence of depression and anxiety within NH/PI communities. For example, one national study found that 36% of NH/PI youth reported past‐year depressed mood versus 26% of White youth [18] while a community‐based study revealed NH/PI adults experience major depression and generalised anxiety disorder at three times and two times the US population rate for these disorders, respectively [19]. This limited data suggests that NH/PIs may be at heightened risk for experiencing significant depression, anxiety and psychological distress during the COVID‐19 pandemic.

### 1.3 NH/PI behavioural health treatment need

Despite NH/PIs' heightened levels of ATOD use, depression and anxiety, few studies have assessed NH/PIs' need for formal behavioural health treatment with the few existing studies indicating that NH/PIs rarely seek formal treatment services unless legally mandated [32, 33]. For example, 76% of NH/PI adults and 67% of young adults reported avoiding/delaying needed treatment services in recent community studies [19, 23]—revealing high levels of unmet NH/PI treatment need before COVID‐19. Consequently, we partnered with NH/PI communities to explore their degree of need *and* unmet need for behavioural health treatment during COVID‐19.

Overall, given NH/PIs' elevated pre‐pandemic rates of ATOD use and mental disorders coupled with strong community concerns regarding COVID‐19's potentially harmful behavioural health impact on NH/PI communities [13], the National Institute on Drug Abuse and National Institute on Alcohol Abuse and Alcoholism funded a large community‐based participatory research investigation of NH/PI ATOD use and treatment need during COVID‐19. In partnership with multiple NH/PI community organisations, this investigation explored the prevalence and relationships of ATOD use, depression, anxiety, COVID‐19‐related distress and need for clinical treatment across diverse NH/PI communities. Obtaining this data would allow clinicians *and* our NH/PI partners to better understand and address the unique substance use and mental health challenges affecting NH/PI communities in the United States—a nation that has suffered among the world's worst COVID‐19 outcomes in infections, hospitalisations and lives lost since March 2020.

## 2 METHODS

### 2.1 Study sample

Research protocols were approved by the University of California, Riverside, Institutional Review Board. NH/PI research staff recruited 306 NH/PI adults (18+ years old) between April and November 2021. As our goal was to obtain a diverse NH/PI community sample across age, gender and ethnicity, our sampling frame contained five states possessing large NH/PI populations: California, Washington, Utah, Oregon and Arkansas.

NH/PIs comprise over 20 ethnic groups hailing from Polynesia, Micronesia and Melanesia. Polynesians (e.g., Native Hawaiian, Samoan) and Micronesians (e.g., Micronesian, Guamanian) are the largest NH/PI populations in the United States, while Melanesians (e.g., Fijian, Papuan) are the smallest. We partnered with NH/PI communities on the continental United States as: (i) health research has historically focused on Native Hawaiians in Hawai‘i, limiting knowledge of the health status/needs of the overall NH/PI population; and (ii) they are the fastest growing US NH/PI communities with over half increasing over 50% in size from 2000 to 2010 [34].

In addition, at the request of our NH/PI partners, we purposively sampled NH/PIs of diverse genders to capture NH/PIs possessing culturally significant non‐binary gender identities. Mirroring the ‘two‐spirit’ gender in some American Indian cultures [35, 36], many NH/PI cultures recognise non‐binary genders such as *mahu*, *fa'afinefine*, *fa'afatama, fokisi* and *fakaleitī* as a distinct gender identities in NH/PI society [37, 38]. Thus, we deliberately oversampled non‐binary NH/PIs to obtain key insights into the behavioural health needs of this understudied group during COVID‐19.

### 2.2 Recruitment

NH/PI staff recruited participants using a rigorous community sampling approach previously demonstrated to reach wide ranges of community individuals. This approach involved stratified multi‐site recruitment including data collection events in NH/PI public spaces, partnering with NH/PI organisations to develop and refine the sampling strategy, and training NH/PI staff from our partner communities to conduct recruitment and data collection [39]. Trained NH/PIs recruited participants via three pathways: personalised e‐mail outreach, telephone calls and in‐person at sites where NH/PIs congregate (e.g., church, recreational centres). Adults were offered two survey options with $20 incentive: online survey via Qualtrics or paper‐and‐pencil survey with measures administered by NH/PI research staff as self‐report or interview (for participants with limited English proficiency). As a community‐based participatory research study, our community partners played a sizable role in determining the recruitment strategies and survey instrument with many of our measures adapted for NH/PIs using feedback from NH/PI research staff, an advisory council of NH/PI experts and cognitive interviews with NH/PI adults (to test clarity and comprehension of study items).

### 2.3 Measures

#### 2.3.1 Demographics and ATOD use

Demographics assessed were age, gender, education, income and marital status. To assess current alcohol use and AUDs, the 3‐item Alcohol Use Disorders Identification Test‐Consumption was used (AUDIT‐C [40]). For current alcohol use, heavy episodic drinking (having six or more drinks per sitting) and possible AUD, the AUDIT‐C asks: (i) ‘How often do you have a drink containing alcohol?’; (ii) ‘How many drinks do you have on a typical day when you are drinking?’; and (iii) ‘How often do you have six or more drinks on one occasion?’ [40]. Higher summed scores indicate higher levels of hazardous drinking with cut‐off scores of ≥4 for men and ≥3 for women indicating probable AUD [40], including in diverse racial/ethnic populations [41]. For non‐binary NH/PIs, we used the non‐gendered Diagnostic and Statistical Manual of Mental Disorders, 5th edition, diagnostic cut‐off score of ≥4 to indicate probable AUD [42]. Sample AUDIT‐C internal consistency was strong (*α* = 0.82).

Participant's lifetime cigarette, e‐cigarette and cannabis use were assessed by asking, ‘*Have you smoked at least 100 cigarettes in your entire life?*’, ‘*Have you ever used any type of e‐cigarette or similar vaping device?’* and ‘*Have you ever, even once, tried marijuana or hashish in any form*?’, while current cigarette, e‐cigarette and cannabis use were assessed by querying, ‘*Do you smoke cigarettes every day, some days, or not at all?*’, ‘*How often, if at all, do you currently use an e‐cigarette or similar vaping device?*’ and ‘*During the past 30 days, on how many days did you use marijuana, hashish, or another THC product*?’ [43, 44, 45].

Lifetime illicit prescription opioid, heroin, and methamphetamine use were ascertained by asking ‘*Have you ever taken prescription pain pills other than ordered by your doctor?*’, ‘*Have you ever taken heroin?*’ and ‘*Have you ever used methamphetamines?’*. For participants who answered ‘yes’ to these lifetime items, to assess current use, they were asked if they had used these substances in the past 30 days. Following federal and international guidelines [46, 47], lifetime illicit substance use was defined as any lifetime cannabis, prescription opioid, heroin or methamphetamine use.

#### 2.3.2 Mental health

Depression and anxiety were assessed using the Patient Health Questionnaire‐9 (PHQ‐9) [48] and Generalised Anxiety Disorder‐7 (GAD‐7) [49], respectively. These measures assessed depression and anxiety symptomology over the prior 2‐week period, possessing excellent validity and reliability with diverse populations [50, 51]. Higher summed scores indicate greater depression and anxiety severity with scores of 10+ indicating major depressive disorder (MDD) and generalised anxiety disorder (GAD), respectively [48, 49]. PHQ‐9 and GAD‐7 internal consistencies were excellent with α's of 0.88 and 0.93, respectively. COVID‐19 distress was measured by asking, ‘*Overall, how much distress have you experienced related to COVID‐19 during the crisis?*’ (1 = Nnone, 10 = Extreme).

#### 2.3.3 Treatment needs

Participants' need for, and delay/avoidance of, behavioural health treatment were assessed using four Medical Expenditure Panel Survey [52] items previously used to assess treatment needs in NH/PI community populations [19, 23]. To determine treatment needs, participants were asked: ‘*In the past 12 months during the pandemic, was there a time when you wanted to talk with or seek help about problems with alcohol, tobacco, cannabis, pain medication, or any other type of substance?*’ and ‘*In the past 12 months during the pandemic, was there a time when you wanted to talk with or seek help about stress, depression, or problems with emotions?*’. To identify delay/avoidance of needed treatment, participants who answered ‘yes’ were asked, ‘*Did you delay or not get the care you thought you needed?*’. Based on prior research showing extremely low NH/PI use of both substance use and mental health treatment [19, 23, 32, 53], we created an aggregated past‐year need for behavioural health treatment variable consisting of ‘yes’ response to the substance use or mental health treatment need items.

### 2.4 Statistical analyses

Data analyses were conducted in SPSS v.27. Frequencies, means and standard deviations were generated using descriptive statistics. Significant gender differences in frequencies and means were analysed using chi‐square and independent *t*‐tests. To address missing data, missing data for prevalence items (e.g., cigarette use) were coded as 0 to signify ‘not present’ and included in the overall prevalence totals while missing data from scales (e.g., PHQ‐9) were scored as 0. Two logistic regression models were created with AUD and any behavioural health treatment need as the dependent variables. Common independent variables across both models were: (i) demographic variables of age, gender, education, income and marital status; and (ii) behavioural health variables of any lifetime illicit substance use, MDD, GAD and COVID‐19 distress. For the treatment need model, AUD was included as an additional independent variable.

## 3 RESULTS

### 3.1 Sample characteristics and ATOD use

Participant characteristics including frequencies of ATOD use and treatment need are detailed in Table 1. Fifty‐five percent of participants self‐identified as women, 32% as men and 13% as non‐binary, with an average age of 35 years.

**TABLE 1:** Characteristics and descriptive statistics for Native Hawaiians/Pacific Islanders participant sample

	Total sample, *N =* 306		Women, *n =* 169		Men, *n =* 96		Non‐binary gender, *n* = 41	
Variables	%	*n*	%	*n*	%	*N*	%	*n*
Formal education								
<High school	11	33	9	16	16	15	5	2
High school graduate	45	134	45	74	52	49	27	11
Some college/college grad	42	125	44	71	29	27	66	27
Prefer not to answer	2	7	2	2	3	3	2	1
Income								
<20,000	24	64	27	40	25	20	11	4
$20,000–$59,999	42	110	40	58	39	32	56	20
$60,000–$100,000+	18	47	17	25	17	14	22	8
Prefer not to answer	16	43	16	24	19	15	11	4
Marital status								
Single/separated/divorced	62	183	61	100	51	47	90	36
Married/living as married	38	114	39	64	49	46	10	4
Separated/divorced	10	30	13	21	10	9	—	—
Ethnicity								
Polynesian	57	168	53	87	51	49	88	36
Micronesian	40	116	43	70	46	44	10	4
Melanesian/other	3	10	4	6	3	3	2	1
Current alcohol use	47	143	34	58	52	50	95	39
Lifetime cigarette use	31	95	21	35	37	35	61	25
Current cigarette use	22	67	13	22	27	26	27	11
Lifetime e‐cigarette use	17	51	9	15	28	13	56	23
Current e‐cigarette use	8	26	6	10	9	9	25	10
Lifetime cannabis use	34	103	28	48	35	34	51	21
Current cannabis use	10	30	8	14	10	10	15	6
Lifetime illicit Rx opioids	6	17	5	8	6	6	7	3
Current illicit Rx opioids	4	11	4	6	3	3	5	2
Lifetime heroin	1	4	2	3	1	1	2	1
Current heroin	1	2	1	2	—	‐	‐‐	‐‐
Lifetime methamphetamines	4	13	2	4	2	2	17	7
Current methamphetamines	1	2	—	—	—	—	5	2
Lifetime illicit substance use	35	108	30	50	35	34	59	24
BH treatment need [past year]	33	102	35	59	22	21	54	22
Avoid/delayed BH treatment	20	61	18	30	14	13	44	18
	**Mean**	**SD**	**Mean**	**SD**	**Mean**	**SD**	**Mean**	**SD**
Age, years	35.13	13.68	36.24	15.72	34.82	11.73	30.66	4.47
Major depression (PHQ‐9)^a^	6.81	6.14	6.80	5.87	6.26	6.55	8.12	6.22
Generalised anxiety (GAD‐7)^b^	5.10	5.73	5.17	5.79	4.40	5.43	6.51	6.01
Alcohol (AUDIT‐C)^c^	2.11	2.73	1.47	2.35	2.52	3.17	3.63	2.27
COVID‐19 distress^d^	4.84	3.34	4.84	3.37	4.22	3.27	6.20	3.02

Abbreviations: BH, behavioural health; Rx, prescription. Lifetime illicit substance use refers to any lifetime cannabis, prescription opioid, heroin, or methamphetamine use.

^a^ Scoring range on the Patient Health Questionnaire‐9 (PHQ‐9) is 0–27. Higher scores indicate greater depressive severity.

^b^ Scoring range on the Generalised Anxiety Disorder‐7 (GAD‐7) is 0–21. Higher scores indicating greater anxiety severity.

^c^ Scoring range on the Alcohol Use Disorders Identification Test–Consumption (AUDIT‐C) is 0–12. Higher scores indicate greater hazardous alcohol use.

^d^ Scoring range on the COVID‐19 Distress scale is 0–10. Higher scores indicate greater COVID‐19‐related psychological distress.

Nearly, half of NH/PI participants reported current alcohol use with higher rates among men versus women (52% vs. 38%; *p* < 0.05). Seventeen percent of participants reported past‐month heavy episodic drinking (having 6 or more drinks per sitting) and 17% consumed 5 or more drinks on a typical drinking day.

Twenty‐seven percent of participants met the original diagnostic cut‐off for probable AUD (AUDIT‐C score ≥4 for men, ≥3 for women). When applying updated Diagnostic and Statistical Manual of Mental Disorders, 5th edition, diagnostic screening cut‐offs for moderate and severe AUD [42], 17% screened positive for severe AUD (AUDIT‐C ≥ 5) and 7% screened positive for moderate AUD (AUDIT‐C ≥ 4).

For lifetime use, approximately one‐third of participants reported lifetime cigarette use with significantly more men versus women (37% vs. 21%; *p* < 0.01). Over one‐third of NH/PIs reported any lifetime illicit substance use. Seventeen percent of participants reported lifetime dual cigarette‐e‐cigarette use and 19% reported lifetime dual cigarette‐cannabis use.

For current use, significantly more men than women currently used cigarettes (27% vs. 13%; *p* < 0.05). Nine percent of participants reported current dual cigarette‐e‐cigarette use and 4% reported current dual cigarette‐cannabis use.

### 3.2 Mental health

Participants possessed high mean depression and anxiety severity scores, with 27% and 19% of participants meeting the diagnostic thresholds for MDD and GAD, respectively. Fifteen percent (*n* = 46) met diagnostic thresholds for both disorders, indicating comorbid MDD and GAD. Nine percent (*n* = 27) and 8% (*n* = 24) of participants met diagnostic thresholds for comorbid AUD with MDD and GAD, respectively.

### 3.3 Behavioural health treatment need

One‐third of NH/PI participants reported needing past‐year behavioural health treatment with significantly more women versus men (35% vs. 22%; *p* < 0.05). Yet, 60% of participants needing treatment reported avoiding/delaying needed treatment. Nine percent (*n* = 27) reported needing both substance use and mental health treatment during COVID‐19.

### 3.4 Non‐binary NH/PIs

Among non‐binary NH/PIs, ATOD use and levels of depression, anxiety and COVID‐19 distress were high. Compared to women and men, non‐binary NH/PIs had the highest rates of lifetime and current use of cigarettes, e‐cigarettes, cannabis, prescription opioids and methamphetamines, with almost 100% reporting current alcohol use—resulting in 46% of non‐binary NH/PIs screening positive for AUD. Non‐binary NH/PIs also had the highest mean severities of depression, anxiety and COVID‐19 distress, and greatest prevalence of MDD (37%) and GAD (24%). Thus, over one‐half of non‐binary NH/PIs reported needing any past‐year behavioural health treatment.

### 3.5 Logistic regressions for AUD and treatment need

For the AUD logistic model (Table 2), being 1‐year older associated with significantly lower likelihood of having AUD (*p* < 0.01), as did having some college/college degree or being married versus having lower than high school education (*p* < 0.05) or being single/unmarried (*p* < 0.05). In contrast, a 1‐point higher level of COVID‐19 distress and engaging in any lifetime illicit substance use associated with greater likelihoods of having AUD (*p* < 0.05).

**TABLE 2:** Logistic regression of alcohol use disorder by demographics, illicit substance use, mental disorders and COVID‐19 distress

	AOR	95% CI
Variables	DV = alcohol use disorder^a^	
Demographic		
Age in years	0.96*	0.93–0.99*
Gender (reference: female)		
Male	1.53	0.77–3.04
Non‐binary	1.93	0.22–1.27
Formal education (reference: <high school)		
High school graduate	0.52	0.22–1.27
Some college/college graduate	0.23*	0.08–0.64*
Income (reference: < $20,000)		
$20,000–$59,999	1.94	0.91–4.14
$60,000–$100,000+	2.36	0.89–6.25
Marital status (reference: single/separated/divorced)		
Married	0.45*	0.21–0.98*
Substance use and mental health		
Lifetime illicit substance use	1.97*	1.02–3.79*
Major depressive disorder^b^	0.78	0.31–1.89
Generalised anxiety disorder^c^	2.38	0.91–6.25
COVID‐19 distress	1.11*	1.01–1.23*

Abbreviations: AOR, adjusted odds ratio; CI, confidence interval; DV, dependent variable.

*p <* 0.01; *p < 0.05.

^a^ Determined via Patient Health Questionnaire‐9.

^b^ Determined via Alcohol Use Disorders Identification Test–Concise.

^c^ Determined via Generalised Anxiety Disorder‐7.

For behavioural health treatment need (Table 3), married participants were less likely to need any treatment versus single/unmarried participants (*p* < 0.01), respectively, while participants with incomes above $20,000 were 2.3–4.8 times more likely to report needing any treatment versus participants making less than $20,000 (*p* < 0.05). A 1‐point higher level of COVID‐19 distress associated with greater likelihood for reporting any behavioural health treatment need (*p* < 0.01) while any lifetime illicit substance use and having MDD associated with 3.0 and 5.3 times greater likelihood of needing treatment (*p* < 0.01), respectively.

**TABLE 3:** Logistic regression of behavioural health treatment need by demographics, illicit substance use, mental disorders and COVID‐19 distress

	AOR	95% CI
Variables	DV = behavioural health treatment need	
Demographic		
Age in years	0.97	0.95–1.01
Gender (reference: female)		
Male	0.48	0.23–1.01
Non‐binary	0.52	0.18–1.46
Formal education (reference: <high school)		
High school graduate	0.89	0.32–2.51
Some college/college graduate	1.42	0.47–4.24
Income (reference: < $20,000)		
$20,000–$59,999	2.36*	1.07–5.10*
$60,000–$100,000+	4.80**	1.82–12.62**
Marital status (reference: single/separated/divorced)		
Married	0.36**	0.17–0.77**
Substance use and mental health		
Lifetime illicit substance use	3.01**	1.49–6.07**
Alcohol use disorder^a^	1.50	0.24–1.89
Major depressive disorder^b^	5.30**	2.18–12.87**
Generalised anxiety disorder^c^	0.46	0.24–1.89
COVID‐19 distress	1.20**	1.07–1.33**

*Note*: *p < 0.01; **p < 0.05.

Abbreviations: AOR, adjusted odds ratio; CI, confidence interval; DV, dependent variable.

^a^ Determined via Alcohol Use Disorders Identification Test–Consumption.

^b^ Determined via Patient Health Questionnaire‐9.

^c^ Determined via Generalised Anxiety Disorder‐7.

## 4 DISCUSSION

In this novel, community‐based participatory research study of diverse US NH/PI communities, we partnered with NH/PI communities to explore community patterns of ATOD use, depression, anxiety, COVID‐19‐related distress and treatment need during the COVID‐19 pandemic. As anticipated, results revealed NH/PI communities experienced high levels of ATOD use, depression, anxiety and unmet treatment needs during COVID‐19.

Notably, over one in four NH/PI adults in our total sample screened positive for AUD; a rate over 2.6 times greater than the 10.2% national AUD rate during COVID‐19 [54]. Analyses indicated that NH/PIs who were younger, unmarried, engaged in lifetime illicit substance use or experienced COVID‐19 distress were more likely to have AUDs. Thus, NH/PIs with these characteristics may benefit greatly from targeted alcohol screening, education and prevention to reduce the heavy alcohol burden affecting NH/PI communities during COVID‐19.

In addition, almost one‐third of NH/PIs reported lifetime cigarette smoking with 22% currently smoking cigarettes—exceeding the 13% US smoking rate during COVID‐19 [55] and mirroring the 23% smoking rate of American Indians/Alaskan Natives; who demonstrate the highest smoking rates of all US racial groups [56]. Because AUDs and smoking generate numerous ‘downstream’ effects including increased violence, accidents, social/legal problems, liver disease and cancer, our findings of high levels of NH/PI AUDs and smoking bolster calls to develop targeted interventions to address NH/PIs' twin alcohol and smoking epidemics [22, 57, 58].

Furthermore, over one‐third of NH/PIs reported lifetime illicit substance use, putting many NH/PIs at heightened risk for addiction and harm over their lifespan [59, 60], However, because NH/PI participants reported similar rates of illicit opioid use and lower methamphetamine use compared to the 2020 US general population rates (3% and 4%, respectively) [54], NH/PIs' risks for lethal and non‐lethal drug overdoses may be lower relative to other US populations. However, given illicit drug use's profound social, psychiatric and economic consequences that may lead NH/PIs who use these substances to be absent from the conventional community locations where NH/PIs congregate (e.g., worksites, cultural organisations/events), it is possible that our surveys may have undercounted the rate of illicit substance use in NH/PI communities. Consequently, to more accurately capture NH/PI opioid and methamphetamine use, future studies should target community locations (bars, homeless encampments) and NH/PI subgroups (persons experiencing homelessness, blue‐collar workers) that possess the greatest risk for NH/PI illicit and polysubstance use.

With regard to mental health challenges, participants also reported heightened depression, anxiety and psychological distress during COVID‐19 with over one‐quarter of NH/PIs screening positive for MDD and one‐fifth for GAD; vastly exceeding the general population rates for these disorders [61, 62]. These current rates also surpassed the elevated MDD, GAD and AUD rates found in pre‐pandemic studies of NH/PIs using the same clinical measures [19, 23]; indicating that NH/PIs likely suffered from increased depression, anxiety and AUDs in response to the COVID‐19 pandemic.

Over one‐third of NH/PI adults also reported needing formal behavioural health treatment during the pandemic, with being unmarried and experiencing COVID‐19 distress associating with greater likelihood of needing treatment. Notably, NH/PIs who engaged in any illicit substance use or had MDD were three to five times more likely to need formal treatment. Yet, because 60% of NH/PIs reporting treatment need avoided/delayed treatment, preventing illicit substance use and alleviating depression and COVID‐19 distress using community‐based approaches (e.g., substance use education, stress coping, support groups, prosocial/cultural activities) may more effectively reduce NH/PI communities' need and unmet need for formal treatment than traditional clinical services.

Lastly, as the first known study to examine behavioural health among non‐binary NH/PIs in the US, it is revelatory that non‐binary NH/PI participants reported exceptionally high levels of ATOD use, depression and anxiety. Across all genders, non‐binary NH/PIs reported the highest prevalence of ATOD use for almost every substance studied plus MDD and GAD. Approximately, one‐half of non‐binary NH/PIs also screened positive for probable AUD and over one‐half reported needing treatment during COVID‐19. Therefore, while our non‐binary sample was relatively small, these findings combined with the higher rates of severe harm (e.g., violence, trauma, suicide) experienced by many gender minorities [63, 64] illuminate the need for further research exploring the behavioural health needs of these at‐risk individuals.

This exploratory study had several limitations including our: (i) inclusion of non‐binary NH/PIs potentially skewing our overall prevalence findings; (ii) use of diverse survey methods that were not accounted for in our analyses possibly affecting participant responses; and (iii) use of non‐probability sampling. Rather, we utilised proven purposive sampling techniques incorporating planned enrolment tables to recruit a diverse sample of NH/PI genders, ages and ethnicities. Based on these study limitations, to confirm our findings, larger population‐based surveys involving additional states (particularly Hawai‘i) should be conducted. In addition, this study did not assess the availability and cultural accessibility of local treatment services. Future studies should therefore seek to explore systemic barriers to NH/PI treatment engagement such as high costs, lack of health insurance and lack of culturally appropriate services; particularly for non‐binary NH/PIs who reported high treatment need but low treatment use.

## 5 CONCLUSION

During the COVID‐19 pandemic, US NH/PIs reported elevated rates of ATOD use, depression and anxiety—placing many NH/PIs in need of effective treatment services. Unfortunately, because NH/PIs rarely seek treatment due in part to a lack of culturally appropriate services [65], the behavioural health repercussions of the COVID‐19 pandemic may affect NH/PI communities for years. Accordingly, culturally responsive prevention, intervention and recovery programs must be developed and implemented to reduce NH/PI substance use and mental health disparities during and after the COVID‐19 pandemic.

## AUTHOR CONTRIBUTIONS

Each author certifies that their contribution to this work meets the standards of the International Committee of Medical Journal Editors. All study protocols were approved by the relevant institutional review board.

## CONFLICT OF INTEREST

The authors have no competing interests to disclose. This project was supported by funding from the National Institute on Drug Abuse [R34 DA049989] and the National Institute on Alcohol Abuse and Alcoholism [R21 AA026689‐S1]. The content is solely the responsibility of the authors and does not represent the official views of the National Institute on Drug Abuse or National Institute on Alcohol Abuse and Alcoholism.

## ETHIC STATEMENT

The research reported here was approved by the University of California, Riverside Institutional Review Board. This statement has now been added to the Methods section.
